# Information Flow Analysis of Interactome Networks

**DOI:** 10.1371/journal.pcbi.1000350

**Published:** 2009-04-10

**Authors:** Patrycja Vasilyev Missiuro, Kesheng Liu, Lihua Zou, Brian C. Ross, Guoyan Zhao, Jun S. Liu, Hui Ge

**Affiliations:** 1 Whitehead Institute for Biomedical Research, Cambridge, Massachusetts, United States of America; 2 Computer Science and Artificial Intelligence Laboratory, Massachusetts Institute of Technology, Cambridge, Massachusetts, United States of America; 3 Dana-Farber Cancer Institute, Harvard Medical School, Boston, Massachusetts, United States of America; 4 Department of Genetics, Washington University, St. Louis, Missouri, United States of America; 5 Department of Statistics, Harvard University, Cambridge, Massachusetts, United States of America; Tufts University, United States of America

## Abstract

Recent studies of cellular networks have revealed modular organizations of genes and proteins. For example, in interactome networks, a module refers to a group of interacting proteins that form molecular complexes and/or biochemical pathways and together mediate a biological process. However, it is still poorly understood how biological information is transmitted between different modules. We have developed *information flow analysis*, a new computational approach that identifies proteins central to the transmission of biological information throughout the network. In the information flow analysis, we represent an interactome network as an electrical circuit, where interactions are modeled as resistors and proteins as interconnecting junctions. Construing the propagation of biological signals as flow of electrical current, our method calculates an *information flow score* for every protein. Unlike previous metrics of network centrality such as degree or betweenness that only consider topological features, our approach incorporates confidence scores of protein–protein interactions and automatically considers all possible paths in a network when evaluating the importance of each protein. We apply our method to the interactome networks of *Saccharomyces cerevisiae* and *Caenorhabditis elegans*. We find that the likelihood of observing lethality and pleiotropy when a protein is eliminated is positively correlated with the protein's information flow score. Even among proteins of low degree or low betweenness, high information scores serve as a strong predictor of loss-of-function lethality or pleiotropy. The correlation between information flow scores and phenotypes supports our hypothesis that the proteins of high information flow reside in central positions in interactome networks. We also show that the ranks of information flow scores are more consistent than that of betweenness when a large amount of noisy data is added to an interactome. Finally, we combine gene expression data with interaction data in *C. elegans* and construct an interactome network for muscle-specific genes. We find that genes that rank high in terms of information flow in the muscle interactome network but not in the entire network tend to play important roles in muscle function. This framework for studying tissue-specific networks by the information flow model can be applied to other tissues and other organisms as well.

## Author Summary

Protein–protein interactions mediate numerous biological processes. In the last decade, there have been efforts to comprehensively map protein–protein interactions occurring in an organism. The interaction data generated from these high-throughput projects can be represented as interconnected networks. It has been found that knockouts of proteins residing in topologically central positions in the networks more likely result in lethality of the organism than knockouts of peripheral proteins. However, it is difficult to accurately define topologically central proteins because high-throughput data is error-prone and some interactions are not as reliable as others. In addition, the architecture of interaction networks varies in different tissues for multi-cellular organisms. To this end, we present a novel computational approach to identify central proteins while considering the confidence of data and gene expression in tissues. Moreover, our approach takes into account multiple alternative paths in interaction networks. We apply our method to yeast and nematode interaction networks. We find that the likelihood of observing lethality and pleiotropy when a given protein is eliminated correlates better with our centrality score for that protein than with its scores based on traditional centrality metrics. Finally, we set up a framework to identify central proteins in tissue-specific interaction networks.

## Introduction

In the last decade, several high-throughput experimental techniques have allowed systematic mapping of protein-protein interaction networks, or interactome networks, for model organisms [1]–[4] and human [5],[6]. Interactome networks provide us with a global view of complex biological processes within an organism. However, it has been a challenge to associate network properties with functional relevance.

Work on global topology of interactome networks has led to a conclusion that these networks are *small-world* with *power-law* degree distributions [7]–[10]. This translates to having a few hub nodes and a majority of nodes with a few partners. This property of interactome networks is very different from random networks where the degree is uniformly distributed. Given that interactomes evolved into this topology, analyzing topological properties of biological networks should provide system-level insights on key players of biological processes.

In an interactome network, the ‘central’ proteins, which topologically connect many different neighborhoods of the network, are likely to mediate crucial biological functions. The most straightforward way of quantifying the centrality of a protein in the context of interactome networks is to examine the protein's degree, e.g. the number of binding partners interacting with the protein of interest. Perturbations of high-degree proteins (hubs) are more likely to result in lethality than mutations in other proteins [7],[11]. However, degree only measures a protein's local connectivity and does not consider the protein's position relative to other proteins except for the direct binding partners of the given protein. A metric to estimate global centrality is *betweenness*. Betweenness determines the centrality of a protein in an interactome network based on the total number of shortest paths going through the given protein [12],[13]. A node partaking in a large fraction of all shortest paths has high betweenness. Such nodes have been termed *bottlenecks* [14] as they are not necessarily high degree (as are the hub nodes), yet they have a large amount of “information traffic.” The bottlenecks, like the hubs, are more likely to be essential than randomly sampled proteins in interactomes [11],[15]. Recent evidence shows that high betweenness is correlated with pleiotropy [16], and bottlenecks tend to mediate crosstalks between functional modules [14].

Both degree and betweenness are graph metrics that are not specifically tailored to describe biological networks. Degree measures a protein's local connectivity and does not consider the protein's position in the network globally. Betweenness is a better measure for centrality in that it takes into account paths through the whole network, but it still has the disadvantage of only considering the shortest paths and ignoring alternative pathways of protein interactions. More importantly, interactome networks can be error-prone and some interactions in the same network are not as reliable as others. Many studies have been conducted to categorize interaction data into different confidence levels [3],[17],[18]. Neither degree nor betweenness takes the confidence levels of interactions into consideration. To provide a better solution for identifying central proteins, we developed an *information flow model* of interactome networks. We took the approach of modeling networks as electrical circuits, which had been presented in previous network analyses [19]–[21]. Construing the propagation of biological signals as flow of electrical current, our method identified proteins central to the transmission of information throughout the network. Unlike the previous methods which characterized only the topological features of proteins, our approach incorporated the confidence scores of protein-protein interactions and automatically considers all possible paths in a network when evaluating the importance of proteins. We compared the information flow score to betweenness, and found that the information flow score in the entire interactome network is a stronger predictor of loss-of-function lethality and pleiotropy, and better tolerates the addition of large amounts of error-prone data.

For a multi-cellular organism, not all interactions have the same propensity to occur in every tissue. However, the current network metrics usually treat interactome networks as a whole, disregarding the possibility that some interactions may not occur at all in certain types of tissues. To address this, we developed a framework for studying tissue-specific networks using the information flow model. We constructed an interactome network for muscle enriched genes in *C. elegans*, and showed that genes of high information flow in the muscle interactome network but not in the entire interactome network are likely to play important roles in muscle function.

## Results

### Information flow model considers interaction confidence scores and all possible paths in protein networks

**Figure 1:**
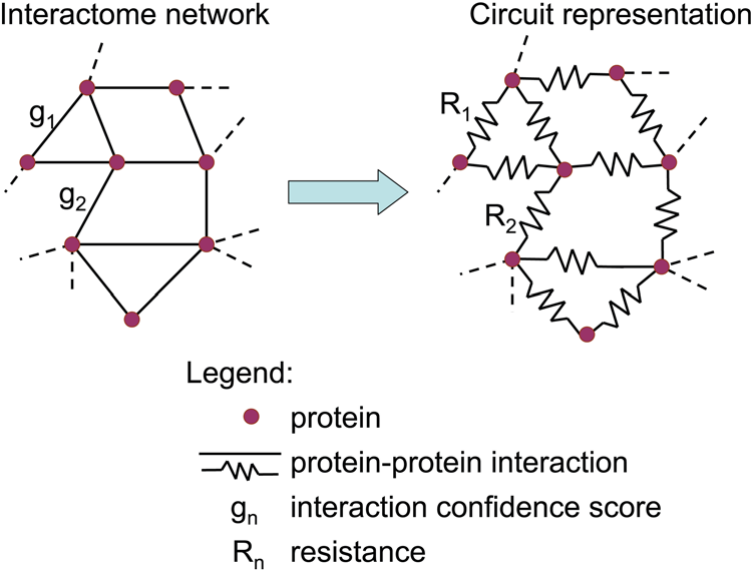
Circuit representation of an interactome network. We model an interactome network as an electrical circuit, where a node represents a protein and a resistor represents an interaction. The resistance value of a resistor is inversely proportional to the confidence score of the corresponding interaction.

We modeled an interactome network as an electrical circuit, where interactions were represented as resistors and proteins as interconnecting nodes (Figure 1). In the circuit, the value of resistance for each resistor is inversely proportional to the confidence score of the interaction. According to Kirchhoff's circuit laws, the current entering any node is equal to the current leaving that node. By applying a current source to one node and grounding another, we determined the exact amount of current flowing through each node in the network (see Materials and Methods). We iterated over all pairwise combinations of “source” and “ground” nodes in the network and summed up the absolute values of current through the node of interest from all iterations. We defined the information flow score of a protein as the sum of absolute values of current through the corresponding node. A node that actively participates in the transmission of current for other nodes ends up with a high sum of absolute values of current, and the corresponding protein receives a high information flow score.

Unlike degree that only considers direct interactions or betweenness that only scores proteins along the shortest paths interpreted as the dominant paths, the information flow model weighs proteins along all the possible paths. Therefore, the information flow model is able to rank “runner-up” proteins participating in many paths of information transmission, instead of only the seemingly prominent ones. This aspect of the information flow model reflects the property of biological pathways more faithfully: there have been plenty of observations for multiple pathways acting in parallel to achieve a specific biological function [22]–[26], and the active pathways may not always be the shortest ones.

We applied the information flow model to two publicly available interactome networks: a *S. cerevisiae* interactome consisting of 1516 proteins involved in 39,099 interactions [3] and a *C. elegans* interactome consisting of 4607 proteins involved in 7850 interactions [2],[27],[28] (see Materials and Methods). Every interaction in the yeast interactome is accompanied by a *socio-affinity index*, which quantifies the tendency for a pair of proteins to identify each other when one of the pair is tagged and to co-purify when a third protein is tagged [3]. A high socio-affinity index indicates a high confidence level for an interaction. We used all the interactions with socio-affinity indices of 2 or higher. The worm interactome does not have numerical scores for the interactions, so we regarded all of the interactions for worms equally. Using these two interactomes, we were able to evaluate the information flow model under situations where interactions are treated equally or interactions have different confidence scores. Similarly to degree and betweenness, information flow scores of proteins in the yeast or worm interactome network did not follow a Gaussian distribution (data not shown), so we converted information flow scores into ranks and percentiles to reflect their relative values in an interactome network.

**Figure 2:**
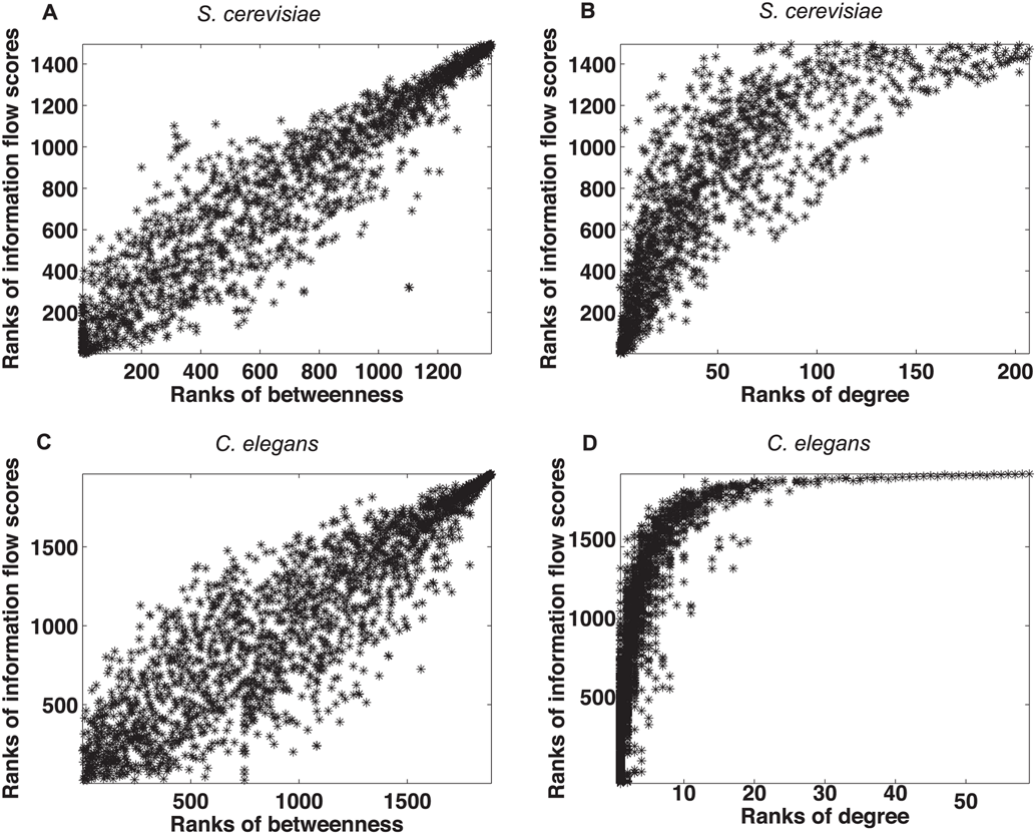
Scatter plots of ranks of information flow versus betweenness (Panel A) or degree (Panel B) in a *S. cerevisiae* interactome network and in a *C. elegans* interactome network (Panel C and Panel D). Overall, ranks of information flow and betweenness are correlated, but a given betweenness usually corresponds to a wide range of information flow scores. Ranks of information flow and degree are less correlated. Low degree can correspond to low, medium or high information flow, but high degree usually corresponds to high information flow.

Although the information flow score is a very different network metric from betweenness or degree, there might be relationships between the information flow score and these two topological metrics. We obtained scatter plots for the ranks of information flow scores versus the ranks of betweenness or degree for both the yeast interactome and the worm interactome (Figure 2). Although the information flow score and betweenness are correlated, a given betweenness rank usually corresponds to a wide range of information flow ranks, and vice versa (Figure 2A and 2C). The information flow score and degree are less correlated (Figure 2B and 2D). Low degree does not necessarily imply low information flow score, although very high degree often implies high information flow score.

### The information flow score is a strong predictor for essentiality and pleiotropy

**Figure 3:**
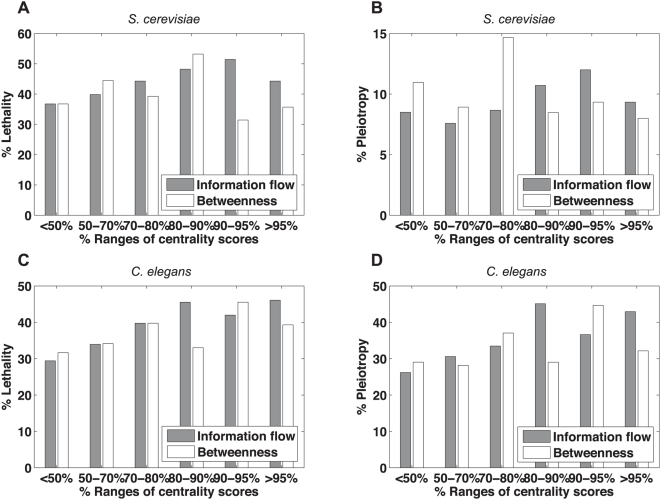
Correlation between information flow scores and loss-of-function phenotypes. The higher a protein's information flow score is, the higher the probability of observing lethality (Panel A) or pleiotropy (Panel B) when the protein is deleted from *S. cerevisiae*. This trend is observed for *C. elegans* as well (Panel C and Panel D). The correlation is not as strong for betweenness and loss-of function phenotypes. The PCCs for information flow scores and phenotypes are 0.84, 0.60, 0.95, and 0.85 in Panels A–D, respectively. In contrast, the PCCs for betweenness and phenotypes are −0.02, −0.31, 0.67, and 0.49 in Panels A–D, respectively.

We propose that the information flow model is able to identify proteins central to the transmission of biological information in an interactome network. If this model works, eliminating the proteins of high information flow scores should be deleterious. The perturbation of information flow and the disintegration of functional modules are likely to result in lethality or multiple phenotypes (pleiotropy). To test our hypothesis, we performed a correlation analysis between the percentages of essential proteins or pleiotropic proteins and the percentiles of information flow scores (see Materials and Methods). For each bin containing proteins within a certain range of information flow scores (in percentiles), we calculated the percentage of proteins whose loss-of-function strains exhibit lethality and the percentage of proteins whose loss-of-function strains exhibit two or more phenotypes. We observed a strong increasing trend for the percentage of essential proteins and the percentage of pleiotropic proteins when information flow scores increase (Figure 3). For *S. cerevisiae*, the Pearson correlation coefficient (PCC) between the percentages of essential proteins and the percentiles of information flow scores is 0.84, and the PCC between the percentages of pleiotropic proteins and the percentiles of information flow scores is 0.60. For *C. elegans*, the PCC between the percentages of essential proteins and the percentiles of information flow scores is 0.95, and the PCC between the percentages of pleiotropic proteins and the percentiles of information flow scores is 0.85 as well.

In contrast, betweenness is a poorer predictor for both essentiality and pleiotropy. For *S. cerevisiae*, the PCC between the percentages of essential proteins and the percentiles of betweenness is −0.02, and the PCC between the percentages of pleiotropic proteins and the percentiles of betweenness is −0.31. For *C. elegans*, the PCC between the percentages of essential proteins and the percentiles of betweenness is 0.67, and the PCC between the percentages of pleiotropic proteins and the percentiles of betweenness is 0.49.

To determine the statistical significance of the correlation, we generated randomized datasets by shuffling genes among the percentile ranges while keeping the number of genes in each range fixed. Next we obtained the percentage of essential or pleiotropic genes for each range and performed correlation analysis for each randomized dataset. We found that the correlation between essentiality or pleiotropy and information flow scores is generally stronger in the actual datasets than in the randomized datasets (*P*-value = 0.0059 and *P*-value = 0.055 for essentiality and pleiotropy in *S. cerevisiae*, respectively; *P*-value = 0.00054 and *P*-value = 0.0047 for essentiality and pleiotropy in *C. elegans*, respectively), while the correlation between essentiality or pleiotropy and betweenness is not significant (P-value>0.05). Information flow outperforms degree in terms of correlation with essentiality or pleiotropy in *S. cerevisiae* (Figure S1). In the *C. elegans* interactome where the interactions are unweighted, degree is still a strong indicator of essentiality and pleiotropy (Figure S1).

### Proteins of high information flow and low betweenness show a high likelihood for being essential or pleiotropic

**Figure 4:**
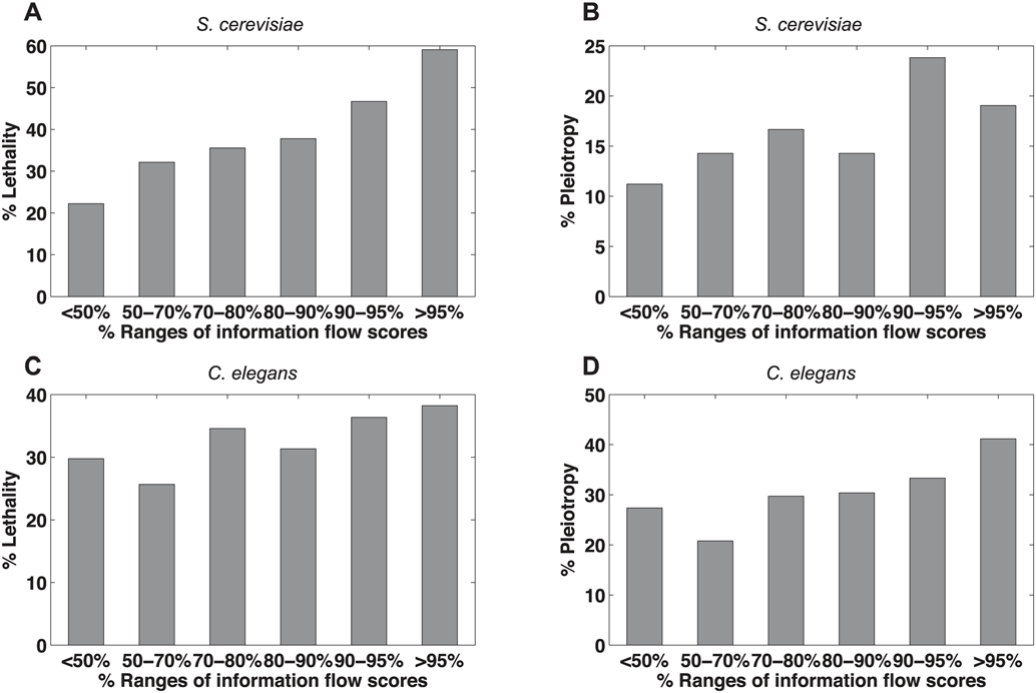
Correlation between information flow scores and loss-of-function phenotypes among proteins of low betweenness. Even among those proteins that rank in the lower 30% in terms of betweenness, a protein's information flow score is still a good indicator for the probability of observing lethality (Panel A) or pleiotropy (Panel B) when the protein is deleted from *S. cerevisiae*. This trend is observed for *C. elegans* as well (Panel C and Panel D). The PCCs for information flow scores and phenotypes are 0.89, 0.79, 0.69, and 0.65 in Panels A–D, respectively.

Proteins with similar betweenness in an interactome can differ significantly in terms of information flow scores (Figure 2). We investigated whether the information flow score is well correlated with essentiality and pleiotropy among proteins that rank low in terms of betweenness. We identified 449 proteins that rank the lowest 30% in the yeast interactome and 672 proteins that rank the lowest 30% in the worm interactome. We found that the correlation between the information flow score and essentiality or pleiotropy holds for these two groups of proteins (Figure 4). For example, we found ten yeast proteins that are among the highest 30% of all proteins in terms of information flow but are among the lowest 30% of all proteins in terms of betweenness. Out of these 10 proteins, 8 correspond to lethal phenotypes when deleted, and the other 2 correspond to multiple other phenotypes when deleted (Table S1). In contrast, we found three yeast proteins that are among the highest 30% of all proteins in terms of betweenness but are among the lowest 30% of all proteins in terms of information flow, and none of them are essential or pleiotropic. Similarly, we found that the information flow model is predictive of essentiality or pleiotropy among medium- or low-degree proteins as well (Figure S2).

What properties make some proteins low in betweenness but high in information flow scores? From the information flow model, we can expect two typical situations: one situation is that a protein lies on alternative paths that are slightly longer than the shortest paths; the other situation is that a protein has a limited number of high-confidence interactions. Betweenness does not take any alternative, longer paths into consideration in the first situation, and betweenness does not give “extra credit” to high-confidence interactions in the second situation. We illustrated the above two situations with example “toy” networks, and analyzed how the information flow model scores nodes that may be important but not recovered by betweenness (Text S1). A closer look at the individual proteins from the interactome networks confirms the existence of both situations in biological networks.

Every interaction in the yeast interactome has a socio-affinity index that measures the likelihood of a true interaction [3]. A hub that has many low-confidence interactions may not be rated as high as a protein with a limited number of high-confidence interactions by the information flow model. We defined an *average interaction score* for a protein as the average of socio-affinity indices for all interactions involving the given protein. For example, SRP68, a core component of the signal recognition particle ribonucleioprotein complex, has a high average interaction score which ranks among the highest 30% in the yeast interactome. SRP68 ranks among the lowest 30% in terms of betweenness but the highest 30% in terms of information flow score. The deletion of this gene results in lethality of the yeast strain. The same situation applies to RPB5, an RNA polymerase subunit. The high average interaction scores are not taken into account in the calculation of betweenness. In the information flow model, we give more credit to the proteins with high-confidence interactions.

**Figure 5:**
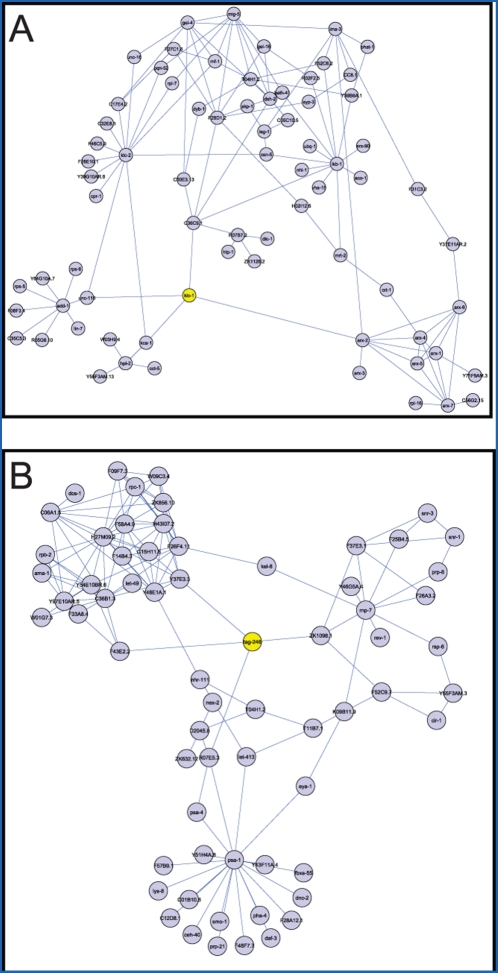
Examples of proteins showing high information flow but low betweenness in the *C. elegans* interactome network. The interactions in the *C. elegans* interactome do not have numerical confidence scores, and the discrepancy between information flow scores and betweenness is likely to be due to topological features such as the existence of alternative paths. KLC-1 (Panel A) and TAG-246 (Panel B) are two worm proteins that have only 4 interactions, and neither of them scores high in betweenness. However, KLC-1 rank the highest 37% and TAG-246 rank in the highest 26% in terms of the information flow scores. The two proteins both correspond to lethal phenotypes upon loss-of-function.

The *C. elegans* interactome does not have numerical scores associated with the interactions, so all the interactions are treated equally in our information flow model. Therefore, the discrepancy of information flow scores and betweenness is likely to result from topological features of the network. For example, KLC-1, which has been found to interact with UNC-116/kinesin, KCA-1/kinesin cargo adaptor, and the ARX-2/Arp2/3 complex component by yeast two-hybrid (Y2H) screens [2], is involved in intracellular transport and is required for embryonic viability. KLC-1 is on a topologically central position (Figure 5A) but scores low in terms of betweenness. Another example is TAG-246, an ortholog of mammalian SWI/SNF-related matrix-associated actin-dependent regulator of chromatin subfamily D (SMARCD). TAG-246 is required for LIN-3/EGF signaling in *C. elegans* vulva development. Just like KLC-1, TAG-246 only has 4 interactions. The loss-of-function of TAG-246 results in lethality as well as several post-embryonic phenotypes, such as *protruding vulva* and *sterile progeny*. Figure 5B shows that there are many parallel paths around TAG-246, so TAG-246 does not always lie on the shortest path, thus scoring low in betweenness. Although KLC-1 and TAG-246 are neither high-degree nor high-betweenness, the information flow model ranks them in the top 37% and top 26%, respectively, because it considers all possible paths in the network.

Taken together, the information flow model is effective in identifying proteins that are central in interactome networks. Even in cases where betweenness ranks are relatively low, the information score serves as a strong predictor for essential or pleiotropic proteins.

### The ranks of information flow scores are more consistent than that of betweenness when a large amount of low-confidence data is added

As more high-throughput datasets become available, new interactions are added into the networks. High-throughput experiments are error-prone and false positives can be problematic [17]. To address the data-quality issue, there have been many studies attempting to estimate the probability of a true interaction between a pair of proteins instead of weighing all interactions equally [18]. However, previous network metrics such as betweenness do not take the likelihood of interactions into account. By incorporating likelihood of interactions into resistor values, the information flow model is able to more accurately simulate information propagation throughout the network.

**Figure 6:**
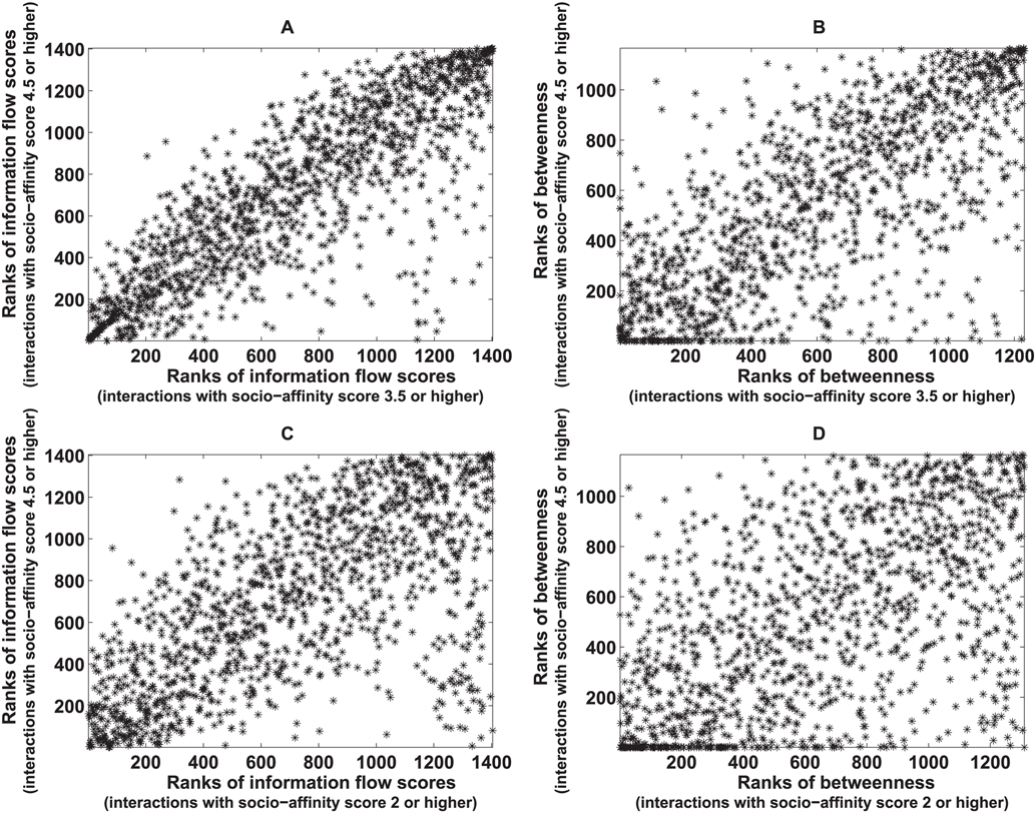
Scatter plots for ranks of information flow scores in different versions of yeast interactome networks (Panel A and C) and for ranks of betweenness in different versions of yeast interactome networks (Panel B and D). The Y-axis represents the rank of information flow scores (Panel A and C) or the rank of betweenness (Panel B and D) in a yeast interactome that includes high-confidence interactions only (socio-affinity scores of 4.5 or higher). In Panel A and Panel B, the X-axis represents the rank of information flow scores or the rank of betweenness in a yeast interactome that includes interactions at lower confidence levels (socio-affinity scores of 3.5 or higher). The PCCs for the ranks of information flow scores (Panel A) and the ranks of betweenness (Panel B) are 0.83 and 0.71, respectively. In Panel C and Panel D, the X-axis represents the rank of information flow scores or the rank of betweenness in a yeast interactome that includes interactions at still lower confidence levels (socio-affinity scores of 2.5 or higher). The PCCs for the ranks of information flow scores (Panel C) and the ranks of betweenness (Panel D) are 0.54 and 0.38, respectively.

In order to analyze how well the information flow model tolerates the addition of a large amount of noisy data, we simulated a growing yeast interactome network by adding low-confidence interactions. Higher socio-affinity indices indicate higher confidence of interactions. In total, there are 9,290 interactions with socio-affinity indices of 4.5 or higher, or 17,159 interactions with socio-affinity indices of 3.5 or higher, or 39,099 interactions with socio-affinity indices of 2 or higher. We rank both information flow scores and betweenness for all the proteins in each of the three versions of the interactome. We showed that ranks of information flow scores were more consistent than that of betweenness when low-confidence interactions were added to the interactome (Figure 6). The consistency of information flow ranks suggests that the information flow model is not only effective but also robust in the case of noise in the data.

### Information flow analysis of a muscle interactome network reveals genes important for muscle function in *C. elegans*

In multi-cellular organisms such as *C. elegans*, a pair of proteins may only interact in certain tissues or cell types. Therefore, the architecture of interactome networks may vary according to tissue or cell types [29]. We hypothesize that proteins of high information flow in a given tissue play crucial roles for the normal function of that tissue.

**Figure 7:**
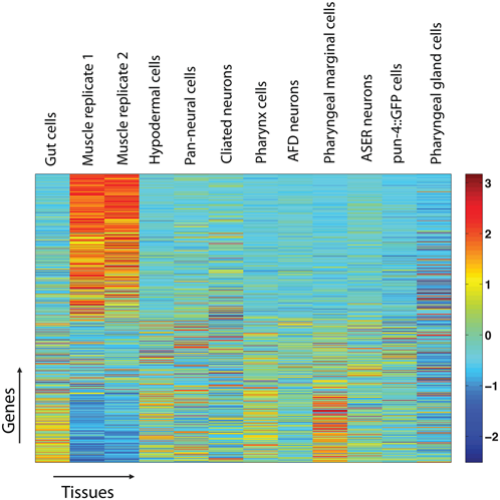
Muscle-enriched genes identified by semi-supervised analysis. Each row represents a gene and each column represents a tissue or cell type. The normalized values of gene expression are represented in a color scale. Genes are sorted by probability scores (P_i_) which indicate expression enrichment in muscle as compared to other tissues. Altogether 310 muscle enriched genes (P_i_≥0.5) were identified. In this plot, the 310 muscle enriched genes, 155 randomly selected genes, and 155 genes with the lowest P_i_ are shown. The list of genes can be found in Table S9.

We tested our hypothesis in an interactome network for muscle-enriched genes. From a SAGE (Serial Analysis of Gene Expression) dataset of 12 *C. elegans* tissues [30], we identified muscle-enriched genes using a semi-supervised learning method [31]. The semi-supervised learning analysis combines the benefits of unsupervised clustering and supervised classification. In other words, both the distribution of data points and prior biological knowledge can be utilized to identify genes enriched in a particular tissue. We manually curated the biomedical literature and found 25 genes known to show enriched expression in muscle cells and 165 genes known not to be expressed in muscle cells (Table S2). These two groups of genes served as positive and negative training data, respectively. For each gene expressed in muscle, the semi-supervised learning procedure gave a probability score (P_i_(muscle)) ranging from 0 to 1 to indicate the gene's expression enrichment in muscle as compared to other tissues (Table S3). We defined genes scoring 0.5 or higher (P_i_≥0.5) as muscle-enriched genes and identified 310 such genes (Figure 7). Among the muscle-enriched genes identified by us, promoter::GFP reporter strains are available for 52 of them, and 31 of them (60%) show clear expression patterns in body wall muscle (Table S4), not including those that might be expressed in other types of muscle. In addition, 260 (84%) of muscle-enriched genes contain *cis*-regulatory modules that indicate expression in muscle in their promoter sequences [32] (Table S5).

From the interactome dataset, we identified direct interacting partners of the muscle-enriched genes. We discarded the interacting genes that, according to the SAGE data, are not expressed in muscle cells. The muscle-enriched genes and their interacting partners which are expressed in muscle form a network of 332 genes and 638 interactions. We defined the weight of an interaction (g_12_) in the muscle interactome network as the product of the probability scores for the two interacting genes (g_12_ = P_1_P_2_). In other words, the more enriched a given gene's expression is in muscle, the higher its propensity is to interact with other enriched genes in muscle cells.

**Figure 8:**
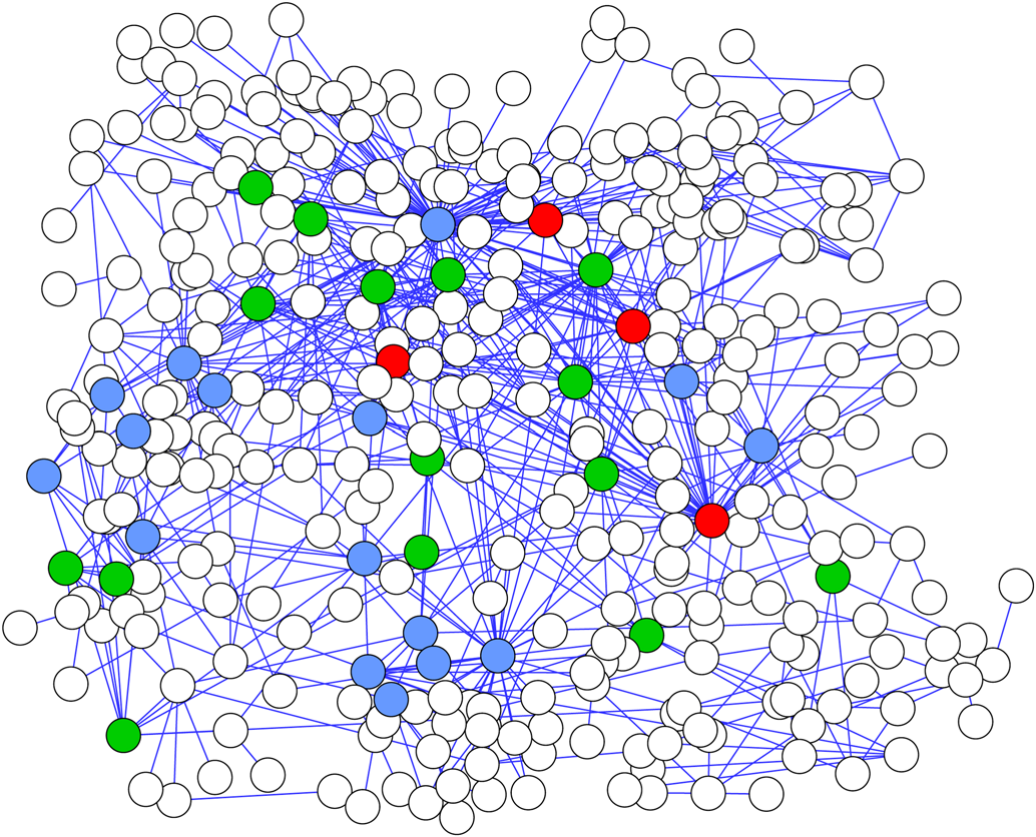
An interactome network for muscle-enriched genes. We identified direct interacting partners for the muscle-enriched genes from the *C. elegans* interactome dataset. We required that an interacting partner must be expressed in muscle cells according to the SAGE dataset. The muscle-enriched genes and their interacting partners form a network. The blue nodes represent the top 20 genes with the highest information flow scores given that the information flow score is calculated just in the muscle network and that the weight of an interaction is defined as the product of the probability scores of the two interacting genes. The green nodes represent the top 20 genes in the muscle network with the highest information flow scores given that the information flow score is calculated in the entire *C. elegans* interactome network and that the interactions are unweighted. Some genes (red nodes) rank in the top 20 under both conditions.

We applied the information flow model to the muscle interactome network, taking the weights of interactions into account. We ranked all the genes in the muscle interactome network by their information flow scores in the muscle interactome network and by their information flow scores in the entire interactome network, respectively. We found that genes of high information flow in the muscle interactome network and genes of high information flow in the entire network did not completely overlap (Figure 8). In other words, some genes rank high in both the muscle network and the entire network, while others rank high in the muscle network but not in the entire network. We first examined genes ranking high in both networks. We identified the top 35 genes based on the sum of their ranks from both networks and found that 40% of them correspond to loss-of-function lethality, which implies that they are essential for the organism development. We then hypothesized that the genes ranking high in the muscle network but not in the entire network play crucial roles in muscle function, though they may not be essential for the whole organism.

We obtained the percentiles of genes in terms of information flow scores in the muscle network and the percentiles of genes in the entire network, calculated the differences between these two percentiles, and ranked the genes by the differences. A *C. elegans* homolog of human paxillin, *tag-327*, shows the largest percentile difference (Table 1). This gene is suspected to be part of the worm muscle attachment complex [33]. A homozygous gene knockout of *tag-327* resulted in uncoordinated animals arrested at the L1 developmental stage, displaying mild disorganization of the myofilament lattice in their muscle cells [33]. The gene showing the second largest percentile difference is *dys-1*, which ranks top 15% in terms of information flow scores in the muscle network and 71% in the entire network. *dys-1* encodes an orthologue of the human DMD [34], which when mutated leads to Duchenne muscular dystrophy, a severe recessive x-linked form of muscular dystrophy that is characterized by rapid progession of muscle degeneration. The gene showing the third largest percentile difference is *lev-11*, which ranks in the top 21% in terms of information flow scores in the muscle network and 78% in the entire network. *lev-11* encodes an orthologue of the human TROPOMYOSIN 1 [35] (www.wormbook.org), which when mutated leads to familial hypertrophic cardiomyopathy, a genetic disorder caused by the thickening of heart muscle. The gene showing the fourth largest percentile difference is *deb-1*, which encodes a muscle attachment protein found in dense bodies, and is required for attaching actin thin filaments to the basal sarcolemma [35] (www.wormbook.org). Out of the top 35 genes that show the largest differences, RNAi feeding strains are available for 25 genes from a library [36]. We performed feeding RNAi experiments using the *rrf-3* strain, an RNAi-sensitive strain, and found that the perturbation of 6 genes (24%) cause motility defect (Table 1). In contrast, RNAi experiments of only 1 out of 16 genes (6%) that rank the lowest in terms of percentile differences revealed any motility defect (Table 1). As a general reference, in a genome-wide RNAi screen using the *rrf-3* strain [37], RNAi experiments of 4.1% of all tested genes showed paralyzed or uncoordinated phenotypes. Even among the muscle-enriched genes identified by the semi-supervised learning method, only 9% of the genes correspond to a paralyzed or uncoordinated phenotype. The analysis' result supports our hypothesis that genes of high information flow specifically in the muscle network play important roles in normal muscle function.

**Table 1:** Genes showing significant difference of information flow scores in the muscle interactome network versus in the entire interactome network.

Gene name	% in the entire interactome network	% in the muscle interactome network	% difference	Motility rate of RNAi-treated worms (thrashes per minute) (mean±s.d.)
*tag-327*	73	14	59	Maternal sterility, unable to score
*dys-1*	72	14	58	103±19
*lev-11*	77	21	56	20±14*
*deb-1*	69	14	55	Maternal sterility, unable to score
F37B4.7	72	21	51	95±30
*dsh-1*	64	13	51	104±22
F41C3.5	66	17	49	105±18
*tag-163*	58	9	49	108±10
*tol-1*	68	25	43	93±26
D2063.1	52	10	42	104±22
Y11D7A.12	45	6	39	113±9
*bath-40*	67	29	38	100±11
*cey-1*	68	32	36	106±13
*lec-2*	59	25	34	111±19
Y62E10A.13	77	45	32	93±10
*unc-87*	34	3	31	16±18*
*unc-15*	35	4	31	12±8*
Y39A1A.3	42	11	31	99±14
*gpd-3*	36	5	31	65±26*
*gly-4*	70	40	30	102±5
*tag-208*	48	18	30	103±11
*uvt-5*	63	33	30	39±30*
*unc-51*	74	45	29	4±9*
*tag-210*	78	49	29	98±10
R07G3.8	73	45	28	93±12
*sec-23*	51	100	−49	102±11
*klc-2*	11	63	−52	48±47*
*pqn-28*	47	100	−53	110±9
M05D6.2	11	63	−52	105±13
*hpl-2*	45	100	−55	110±8
F14E5.2	44	100	−56	Maternal sterility, unable to score
*unc-84*	43	100	−57	104±11
*lap-1*	40	100	−60	104±6
F11D5.1	39	100	−61	111±12
*ttm-1*	36	100	−64	105±13
*emb-30*	30	100	−70	100±12
F31E3.2	30	100	−70	115±8
*tag-205*	16	100	−84	97±15
T18D3.7	15	100	−85	111±7
*lrx-1*	12	100	−88	114±12
*sta-1*	12	100	−88	114±9

The normal motility of the *rrf-3* strain is 99±8 thrashes per minute. Genes with * show significantly lower motility rates upon RNAi treatment compared to the *rrf-3* strain.

It is plausible that the genes showing higher information flow scores in the muscle network than the entire network can also be distinguished by conventional methods such as betweenness. To clarify this, we obtained the percentiles of genes in terms of betweenness in the muscle network and that of genes in the entire network, and ranked the genes by the differences between the two percentiles (Table S6). The top genes identified by differences in information flow do not necessarily rank high by the differences in betweenness (Table 1 and Table S6). For example, *tag-327*, *dys-1*, *lev-11*, and *deb-1*, the top four genes identified by differences in information flow, only rank No. 20, 23, 58, and 59 by differences in betweenness, respectively. This is due to the fact that the information flow model considers the confidence of interactions derived from co-expression while betweenness does not. Similarly, if we rank genes by the probability of expression in muscle, P_i_(muscle), as derived from the semi-supervised learning method, *tag-327*, *dys-1*, *lev-11*, and *deb-1* rank only at No. 149, 269, 97, and 124, respectively. The relevance in muscle function of these genes has been reported in the literature [33]–[35], suggesting that the information flow method does identify biologically relevant candidate genes that can be distinguished using neither the gene expression data nor a graph metric such as betweenness.

## Discussion

We model interactome networks as large electrical circuits of interconnecting junctions (proteins) and resistors (interactions). Our model identifies candidate proteins that make significant contributions to the transfer of biological information between various modules. Compared to degree and betweenness, our model has two major advantages: first, it incorporates the confidence scores of protein-protein interactions; second, it considers all possible paths of information transfer. When a protein that mediates information exchange between modules is knocked down, the disintegration of multiple modules is very likely to result in lethality. Even if the organism is still viable, pleiotropy may be observed because multiple phenotypes imply the breakdown of multiple modules. In support of our model, we find that the information flow score of a protein is well correlated with the likelihood of observing lethality or pleiotropy when the protein is eliminated. Even among proteins of low or medium betweenness, the information flow model is predictive of a protein's essentiality or pleiotropy. Compared to betweenness, the information flow model is not only more effective but also more robust in face of a large amount of low-confidence data. Our method is accessible to the public. The MATLAB implementation of the information flow algorithm, along with the information flow scores for proteins in the yeast interactome network and proteins in the worm interactome network, can be downloaded at http://jura.wi.mit.edu/ge/information_flow_plos/.

**Figure 9:**
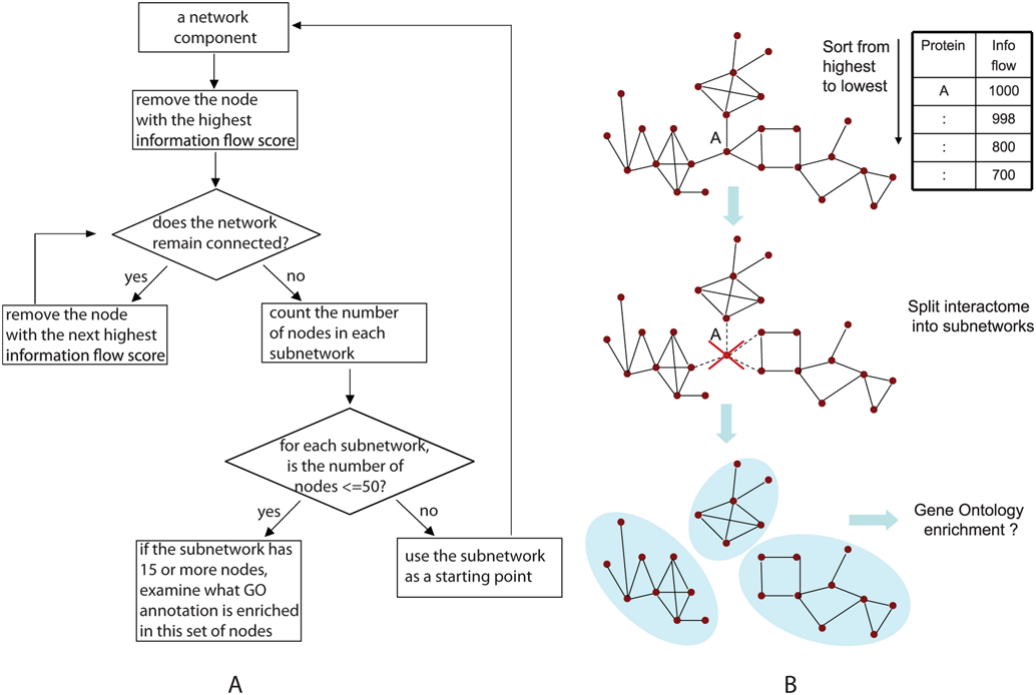
An interactome network can be partitioned into subnetworks by recursively removing proteins of high information flow scores. Panel (A) shows our procedure for network partition, and Panel (B) shows a “toy” example.

The information flow model identifies central proteins in interactome networks, and these proteins are likely to connect different functional modules. We developed an algorithm that decomposes interactome networks into subnetworks by removing proteins of high information flow in a recursive manner (Figure 9) (Materials and Methods). Starting from the largest network component, we removed the protein with the highest information flow score. If the proteins remained connected in a single network, we removed the protein with the next highest information flow score one-at-a-time, until the network fell into multiple pieces upon the protein removal. We then counted the number of proteins in each of the subnetworks. If a subnetwork contained between 15 and 50 proteins, we examined whether any Gene Ontology (GO) term was enriched among proteins in the subnetwork [38],[39]. If a subnetwork contained over 50 proteins, we repeated the procedure of removing high information flow proteins from the subnetwork. Overall, we obtained 37 subnetworks, and all but two of them were enriched with proteins from certain GO categories (Table S7). We investigated the effects of varying the minimum and maximum size of subnetworks (Text S2). The selected range of 15 to 50 proteins was based on the number of recovered subnetworks as well as the overall GO enrichment scores. If we increased the minimum subnetwork size to 20 proteins, the number of subnetworks shrank to 24, all of which were functionally enriched. However, in order to recover the additional 11 GO enriched subnetworks for a total of 35, we decided to keep the lower threshold at 15 proteins. The fact that the majority of subnetworks are functionally enriched provides additional evidence that proteins with high information flow score interconnect different modules.

It was previously observed in a yeast interactome network that ‘date hubs’, which connect different modules, are more likely to participate in genetic interactions than randomly sampled proteins, because elimination of date hubs may make the organism more sensitive to any further genetic perturbations [40]. We tested whether proteins of high information flow and proteins of high betweenness show the same property in the *C. elegans* interactome. We found that genes that rank the highest 30% in terms of information flow or betweenness are more likely to participate in genetic interactions than randomly selected genes (*P*-value = 1.16×10^−10^ and *P*-value = 1.16×10^−10^, respectively). This is not particularly surprising because many proteins of high information flow or high betweenness are hubs in the network.

Another possible feature of “between-module” proteins is related to the expression dynamics of these proteins and their interacting partners. In general, interacting proteins are likely to share similar expression profiles [41]. Date hubs in yeast interactome networks have been found to be less correlated with their binding partners in terms of expression dynamics than ‘party hubs’ which function within a functional module [40]. Proteins of high betweenness in yeast interactome networks have also been reported to show the lack of expression correlation with their binding partners [14]. On the other hand, it has been argued in another study that the lack of correlation is dependent on the datasets examined [42]. We investigated the correlation of expression profiles [43],[44] for proteins of high information flow or proteins of high betweenness with their interacting partners in the *C. elegans* interactome. We did not find proteins of high information flow or proteins of high betweenness behaving differently from other proteins in terms of expression correlation with their interacting partners (data not shown). Thus the expression correlation between topologically central proteins and their binding partners may be worth further investigations.

The transmission of biological signals is directional while at present interactome networks often reflect the formation of protein complexes [3] and do not contain directionality. We explored whether the information flow model is also applicable to signaling networks with directionality. We generated a signaling network for *S. cerevisiae* by integrating phosphorylation events [45] and Y2H interactions (see Materials and Methods). In this network, we examined the top 30% versus the bottom 30% of genes ranked by the information flow score. We found a significant increase in the percentage of pleiotropic genes in the former group (17.0%) as compared to the latter (5.3%) (Table S8) (*P*-value = 0.01), though the percentages of essential genes are similar for the two groups. This analysis suggests that the information flow model is useful for discovering crucial proteins in signaling networks, as well as in networks of protein complexes. The lack of correlation with lethality may reflect the fact that fewer proteins in signaling networks participate in “housekeeping” functions, which are often mediated by multi-protein molecular machines.

In the future, with more information integrated into interactome networks, we should be able to improve on the performance of information flow model. In addition, interactome networks can vary at different times or in different spatial locations. After all, we still have very limited understanding of how biological information flows through cellular networks. Most likely, it does not flow exactly as the electrical current flow does. As more knowledge is accumulated, we should be able to modify the information flow model according to the design principles of cellular network and highlight the dynamic nature of cellular networks.

## Materials and Methods

### Data sources

All of the data used in our study comes from openly available databases and published high-throughput datasets. We obtained a list of essential genes for *S. cerevisiae* from the *Saccharomyces* Genome Database (http://www.yeastgenome.org/) and a list of essential genes for *C. elegans* embryos from the WormBase (http://www.wormbase.org/). We downloaded phenotypic data of *S. cerevisiae* deletion strains under various conditions [46] and *C. elegans* post-embryonic phenotypes from genome-wide RNAi screens [37],[47]. We also downloaded interaction datasets for *S. cerevisiae* [3],[45],[48] and *C. elegans* [2],[26],[27].

### Betweenness

Betweenness is a centrality measure of a node in a network graph. The betweenness of a particular node is determined by how often it appears on the shortest paths between the pairs of remaining nodes [12]. For a graph with *N* nodes, the betweenness *C_B_*(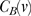) for node 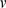 is:
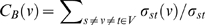
where *σ_st_* represents the number of shortest paths from node *s* to node *t*, and *σ_st_(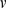)* represents the number of shortest paths from node *s* to node *t* that pass through node 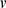. To compute shortest path, we used Dijkstra algorithm [49]. Dijkstra algorithm is a greedy search algorithm that solves the single-source shortest path problem for a directed graph with non negative edge weights. We modified it to handle edges without directionality.

### The information flow model

We model an interactome network as a resistor network, where proteins are represented as nodes and interactions are represented as resistors. The conductance of each resistor is directly proportional to the confidence score of the corresponding interaction. In cases where the confidence levels of interactions are not known, we assume that all resistors have unit conductance.

In order to estimate the importance of node *k* in conducting electrical current in a network of N nodes, we connect node *i* to a unit current source and node *j* to the ground, and we compute how much current flows through node *k* using Kirchhoff's laws (see Figure S3). We define the information flow score of node *k* as the sum of current through node *k* among all pair-wise combinations of source and ground nodes. Since exchanging the source node and the ground node does not lead to different current distributions, we perform the calculation of information flow scores only for cases where *i*>*j*. The total number of pair-wise combinations of nodes (*i*,*j*), such that *i*≠*k*, *j*≠*k* and *i*>*j* is (N-2)(N-3)/2. The information flow through node *k* is
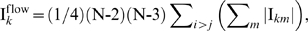
(1)where I*_km_* is the current between the nodes *k* and *m*, and Σ*_m_* is the sum over all resistors connected to node *k*.

For a given pair of source node and ground node, the standard way of computing resistor currents of a circuit is using *nodal analysis* and solving the resulting system of (*N* -1) linear equations for node voltages. For each node *m* that is not a ground node, we have the following equation:
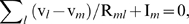
(2)where v*_l_* is a voltage at node *l*, and the sum is over all nodes directly connected to node *m*. When node *m* is a source node, the right-hand side of equation (2) is a unit value of current. Node voltages can be computed by solving the following linear system of equations:
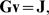
(3)where **G** is a symmetric (*N*-1)×(*N*-1) conductance matrix, **v** is a vector of unknown node voltages and **J** is a vector of currents to every node. The matrix **G** can be calculated using the following algorithms.

#### Algorithm 1: assembly of the nodal matrix

1. Initialize an *N*×*N* matrix G^*^ to zero.
2. For every resistor in the circuit:
  - Insert the off-diagonal element g*_ij_* = g*_ji_* = (−1/R*_ij_*), where *i* and *j* are the end terminals of the resistor;
  - Add the value (1/R*_ij_*) to both diagonal values g*_ii_* and g*_jj_*.
3. Remove the row and column of G^*^ corresponding to the ground node (since its voltage is zero).

The right-hand-side of the equation (3) is a vector of currents, which is zero except for the source node *i* which has a unit value. The most time consuming part of solving (3) is LU decomposition of matrix **G**. Since **G** remains the same if the ground node is fixed, we can reuse matrices **L** and **U** while iterating over all source nodes. Therefore, we need only *N* LU decompositions of **G**.

Below we outline the resulting algorithm for calculating information flow of a given circuit.

#### Algorithm 2: calculation of the information flow

1. Assemble the *N*×*N* matrix G^*^ by following steps 1 and 2 of the Algorithm 1.
2. Initialize the absolute sum of currents for each node to be the zero vector **I_Σ_**.
3. Iterate over the ground node *j* = 1…*N*:
  - Get matrix **G** by removing the row and column *j* of G^*^ (step 3 of algorithm 1);
  - Compute the LU decomposition of matrix **G**.:
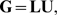
where **L** is lower-diagonal matrix and **U** is upper-diagonal;
  - Iterate over the source node *I* = (*j*+1)…*N*:
    1. Set the right-hand-side vector **J** to have all zeros except the unit i*^th^* entry;
    2. Solve for node voltages **v** using matrices **L** and **U**:
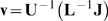

    3. Compute the absolute sum of all currents for each node and add them to the entries of **I_Σ_**.
4. Using (1), compute the information flow for each node.

### Decomposition of interactome networks using the information flow model

Our information flow model identifies central proteins in interactome networks. Very likely the proteins of high information flow scores represent connecting points of functional modules. To test this hypothesis, we designed an algorithm to recursively remove the highest flow proteins and release subnetworks from a large interactome network component. In the algorithm described below, a ‘core module’ refers to a subnetwork composed of 15 to 50 proteins.

#### Algorithm: recursive node removal

1. Initialize:
  - core module set, M*, to an empty set,
  - core module size limits, *s_min_ = 15* and *s_max_ = 50*,
  - **G** to the set of all genes;
  - G* to the set of all proteins sorted from highest to lowest information flow score;
  - **R**, the set of proteins that have been removed, to an empty set,
  - **C**, the protein connectivity matrix, with a 1 for each protein-protein interaction and 0 s for no interaction.
2. Iterate while **G** is not empty:
  - Given **G** and **C**, extract a list of protein modules, **S**.
  - Initialize nodes to be removed from **G**, **G_remove_** to an empty set.
  - Iterate over the set of modules **S**, *i = 1 … size(* ***S*** *)*:
    1. If number of genes in **S**(i), *size(* ***S*** *(i))< = s_max_*
      1. If *size(* ***S*** *(i))> = s_min_*
        Append ***S*** *(i)* to M*,
      2. Add genes in ***S*** *(i)* to **G_remove_**
  - Remove nodes present in **G_remove_** from **G**.
  - Initialize high flow node(s) to be removed at this iteration, **F**, to an empty set.
  - Iterate while G* is not empty and **F** is empty
    1. Remove next highest flow protein(s) from G* and assign it to **F**,
    2. Set **F** to nodes common to **G** and **F**,
    3. Append **F** to **R**.

### Applying information flow model to a yeast signaling network

To evaluate the performance of information flow in signaling networks, we combined a phosphorylation dataset for *S. cerevisiae* which contained kinases and their target proteins [45] with various sources of Y2H data [48]. Specifically, we searched for Y2H interactions between the target proteins in the phosphorylation dataset. As a result, we obtained a set of 77 kinases involved in 1008 phosphorylation events with 312 target proteins interconnected by 503 Y2H interactions. Each kinase phosphorylates one or more of the 312 proteins in the Y2H network. In order to retain the directionality of phosphorylation in the information flow model, we compute the information flow separately for each kinase. First, we use directed edges to link the kinase to its phosphorylation targets in Y2H network. Next, we set the kinase to be a source and sequentially set the remaining 312 proteins to be sinks as we compute the information flow. Before we move on to the next kinase, we remove the phosphorylation edges specific to the previous kinase. The total information flow score for each of the 312 proteins in the Y2H network is obtained by summing the absolute values of information flow from 77 kinase-specific networks.

### RNA interference

We performed RNA interference (RNAi) experiments by feeding L4 worms, following protocols from the WormBook [50] (www.wormbook.org). The bacteria strains for feeding RNAi experiments were from an RNAi library [36] that is commercially available.

## Supporting Information

Figure S1 Correlation between degrees and loss-of-function phenotypes. The higher a protein's degree is, the higher the probability of observing lethality (Panel C) or pleiotropy (Panel D) when the protein is deleted from *C. elegans*. However, this trend is not observed for *S. cerevisiae* (Panel A and Panel B). The PCCs for degrees and phenotypes are 0.31, −0.53, 0.96, and 0.97 in Panels A–D, respectively.
(0.08 MB DOC)

Figure S2 Correlation between information flow scores and loss-of-function phenotypes among proteins of low or medium degrees. Even among proteins of low or medium degrees, a protein's information flow score is still a good indicator for the probability of observing lethality (Panel A) or pleiotropy (Panel B) when the protein is deleted from *S. cerevisiae*. This trend is observed for *C. elegans* as well (Panel C and Panel D). The correlation is not as strong for betweenness and loss-of function phenotypes. The PCCs for information flow scores and phenotypes are 0.80, 0.86, 0.84, and 0.80 in Panels A–D, respectively. In contrast, the PCCs for betweenness and phenotypes among low- or medium-degree proteins are 0.61, 0.037, 0.32, and 0.49 in Panels A–D, respectively.
(0.10 MB DOC)

Figure S3 Kirchhoff's Current Law: the basis for calculating information flow scores.
(0.10 MB EPS)

Table S1 Genes in the *S. cerevisiae* interactome that rank the highest 30% by information flow and rank the lowest 30% by betweenness.
(0.02 MB DOC)

Table S2 Training examples in the semi-supervised analysis of genes expressed in *C. elegans* muscle cells.
(0.02 MB XLS)

Table S3 A list of genes expressed in *C. elegans* muscle cells with their probability scores.
(0.71 MB XLS)

Table S4 A list of muscle-enriched genes for which promoter::GFP strains are available.
(0.03 MB XLS)

Table S5 A list of muscle-enriched genes identified by the semi-supervised analysis. We scored whether the promoters of these genes contain cis-regulatory modules that indicate gene expression in muscle.
(0.04 MB XLS)

Table S6 Genes showing significant difference in betweenness scores in the muscle interactome network versus in the entire interactome network.
(0.04 MB XLS)

Table S7 Subnetworks revealed by recursive removal of genes of high information flow from the *C. elegans* interactome. Only the subnetworks that contain 15 to 50 genes are shown. The P-value cutoff for enrichment of Gene Ontology terms is set at 0.1. If multiple Gene Ontology terms are enriched in a subnetwork, only three of them are displayed in this table.
(0.07 MB DOC)

Table S8 Information flow scores, lethality, and pleiotropy scores of proteins which are part of a signaling network for *S. cerevisiae* containing phosphorylation binding events and Y2H interactions.
(0.03 MB XLS)

Table S9 A list of genes shown in Figure 7. This list includes 310 genes with enriched expression in muscle cells, 155 randomly selected genes, and 155 genes that are the least likely to be enriched in muscle as identified by the semi-supervised analysis.
(0.04 MB XLS)

Text S1 In order to better illustrate the properties of information flow which are not exhibited by betweenness, we analyze two toy examples of possible network topologies using either of the two methods.
(0.05 MB DOC)

Text S2 We executed the module extraction routines while varying the maximum and the minimum number of proteins allowed in a single subnetwork in order to determine the best size range.
(0.06 MB DOC)
