# Supplementary material for: Information Flow Analysis of Interactome Networks
Source: PLoS Comput Biol. 2009 Apr 10;5(4):e1000350. doi: 10.1371/journal.pcbi.1000350 (PMC2685719; doi:10.1371/journal.pcbi.1000350)
Supplement: Figure S1 — Correlation between degrees and loss-of-function phenotypes. The higher a protein's degree is, the higher the probability of observing lethality (Panel C) or pleiotropy (Panel D) when the protein is deleted from C. elegans. However, this trend is not observed for S. cerevisiae (Panel A and Panel B). The PCCs for degrees and phenotypes are 0.31, −0.53, 0.96, and 0.97 in Panels A–D, respectively. (0.08 MB DOC) [file pcbi.1000350.s001.doc]

Figure S1. Correlation between degrees and loss-of-function phenotypes. The higher a protein’s degree is, the higher the probability of observing lethality (Panel C) or pleiotropy (Panel D) when the protein is deleted from *C. elegans*. However, this trend is not observed for *S. cerevisiae* (Panel A and Panel B). The PCCs for degrees and phenotypes are 0.31, -0.53, 0.96, and 0.97 in Panels A-D, respectively.
