# Supplementary material for: Information Flow Analysis of Interactome Networks
Source: PLoS Comput Biol. 2009 Apr 10;5(4):e1000350. doi: 10.1371/journal.pcbi.1000350 (PMC2685719; doi:10.1371/journal.pcbi.1000350)
Supplement: Figure S2 — Correlation between information flow scores and loss-of-function phenotypes among proteins of low or medium degrees. Even among proteins of low or medium degrees, a protein's information flow score is still a good indicator for the probability of observing lethality (Panel A) or pleiotropy (Panel B) when the protein is deleted from S. cerevisiae. This trend is observed for C. elegans as well (Panel C and Panel D). The correlation is not as strong for betweenness and loss-of function phenotypes. The PCCs for information flow scores and phenotypes are 0.80, 0.86, 0.84, and 0.80 in Panels A–D, respectively. In contrast, the PCCs for betweenness and phenotypes among low- or medium-degree proteins are 0.61, 0.037, 0.32, and 0.49 in Panels A–D, respectively. (0.10 MB DOC) [file pcbi.1000350.s002.doc]

Figure S2. Correlation between information flow scores and loss-of-function phenotypes among proteins of low or medium degrees. Even among proteins of low or medium degrees, a protein’s information flow score is still a good indicator for the probability of observing lethality (Panel A) or pleiotropy (Panel B) when the protein is deleted from *S. cerevisiae*. This trend is observed for *C. elegans* as well (Panel C and Panel D). The correlation is not as strong for betweenness and loss-of function phenotypes. The PCCs for information flow scores and phenotypes are 0.80, 0.86, 0.84, and 0.80 in Panels A-D, respectively. In contrast, the PCCs for betweenness and phenotypes among low- or medium-degree proteins are 0.61, 0.037, 0.32, and 0.49 in Panels A-D, respectively.
