# Supplementary material for: Information Flow Analysis of Interactome Networks
Source: PLoS Comput Biol. 2009 Apr 10;5(4):e1000350. doi: 10.1371/journal.pcbi.1000350 (PMC2685719; doi:10.1371/journal.pcbi.1000350)
Supplement: Table S1 — Genes in the S. cerevisiae interactome that rank the highest 30% by information flow and rank the lowest 30% by betweenness. (0.02 MB DOC) [file pcbi.1000350.s004.doc]

Table S1. Genes in the *S. cerevisiae* interactome that rank the highest 30% by information flow and rank the lowest 30% by betweenness.

| Gene Name | Lethality? | Number of phenotypes other than lethality |
| --- | --- | --- |
| SRP68 | Yes |  |
| RPB5 | Yes |  |
| PAP2 | No | 2 |
| RPB8 | Yes |  |
| RRP4 | Yes |  |
| LSM2 | Yes |  |
| MRPL4 | No | 13 |
| PRP31 | Yes |  |
| NOP14 | Yes |  |
| NOP7 | Yes |  |
