# Supplementary material for: Information Flow Analysis of Interactome Networks
Source: PLoS Comput Biol. 2009 Apr 10;5(4):e1000350. doi: 10.1371/journal.pcbi.1000350 (PMC2685719; doi:10.1371/journal.pcbi.1000350)
Supplement: Table S7 — Subnetworks revealed by recursive removal of genes of high information flow from the C. elegans interactome. Only the subnetworks that contain 15 to 50 genes are shown. The P-value cutoff for enrichment of Gene Ontology terms is set at 0.1. If multiple Gene Ontology terms are enriched in a subnetwork, only three of them are displayed in this table. (0.07 MB DOC) [file pcbi.1000350.s010.doc]

Table S7. Subnetworks revealed by recursive removal of genes of high information flow from the *C. elegans* interactome. Only the subnetworks that contain 15 to 50 genes are shown. The P-value cutoff for enrichment of Gene Ontology terms is set at 0.1. If multiple Gene Ontology terms are enriched in a subnetwork, only three of them are displayed in this table.

| No. | Number of genes | List of genes | Gene Ontology enrichment |
| --- | --- | --- | --- |
| 1 | 15 | C31E10.7, nhr-129, mif-3, tag-318, F29A7.3, srv-5, end-3, K08B5.2, rnp-4, mag-1, nas-22, T21H8.5, T27A8.2, app-1, str-173 | macromolecule metabolic process; nucleus; germline sex determination |
| 2 | 17 | B0034.1, C36H8.1, cyc-1, C55C3.1, egl-21, far-2, npp-17, cup-2, F27C1.4, isp-1, drr-1, K02B7.3, col-101, M01F1.4, Y23H5A.4, Y38C1AA.7, Y38F1A.6 | mitochondrial electron transport chain/respiratory chain |
| 3 | 22 | AH6.2, C28G1.3, sec-10, C47D2.1, D2030.1, grl-24, F30F8.3, F31E8.4, F39B2.7, syp-3, F52H3.2, F54D10.5, opt-3, nsy-1, pme-4, grp-1, R06C7.5, lin-59, sos-1, taf-7.2, ZK945.7, ZK973.11 | oviposition; membrane docking; tRNA processing |
| 4 | 16 | C06E4.2, C32D5.11, F20G4.1, F23H11.3, F33D4.6, mec-8, him-10, R74.5, etr-1, adr-2, sup-12, VW02B12L.4, ndc-80, W02A11.3, Y34B4A.6, Y59A8B.10 | RNA binding; organ morphogenesis; response to stimulus |
| 5 | 23 | B0280.3, mcm-3, smn-1, C50B8.4, F02H6.3, F10B5.2, mcm-7, F41H10.4, F48E3.8, F54D11.4, F55F10.1, K04G2.6, npp-1, cyk-4, rfp-1, mcm-5, T24D5.3, mua-6, mrg-1, hpr-9, ZK1127.3, ZK507.6, mcm-6 | DNA replication initiation; cytokinesis; nucleus |
| 6 | 19 | tag-188, C09G12.9, rnf-5, C25D7.8, CD4.4, npp-5, nhr-108, F35F10.12, ubc-20, cyp-31A2, frm-8, taf-10, let-70, R06B9.3, drp-1, ubc-26, ubc-16, ubc-14, ubc-8 | ubiquitin cycle; ligase activity; biopolymer metabolic process |
| 7 | 15 | prk-1, C18E3.9, C34D4.2, tag-325, mlc-4, F21H7.3, tag-204, src-2, tag-218, R09B3.2, T10E10.4, pen-2, col-181, nhr-251, ZK938.1 | none |
| 8 | 27 | bas-1, ceh-33, vha-16, D1054.1, sgn-1, F13E6.5, vha-12, vha-10, ina-1, vha-14, bli-4, mup-4, mua-3, R07E5.7, vha-2, vha-4, vha-15, lrp-2, col-3, W08E12.7, vha-11, vha-3, ttm-1, Y48B6A.1, pat-3, unc-32, vha-9 | proton transport; ATP biosynthetic process; larval development |
| 9 | 20 | rps-29, C17G10.2, C24A3.2, trs-1, E02H1.6, klp-13, rps-23, rps-15, rps-14, rps-11, F41E7.3, rps-22, F58B3.4, rps-16, rps-5, T09B4.8, rps-20, rps-4, lad-2, rps-18 | ribosome; translation; larval development |
| 10 | 15 | nhr-10, C06A8.1, pas-6, F09E5.7, F42A10.5, K04G2.10, lin-31, eif-3.D, lin-46, T07F8.4, T08A11.1, Y39G10AR.15, Y82E9BR.13, ZK930.3, pas-7 | proteasome core complex; sex differentiation; positive regulation of growth |
| 11 | 20 | casy-1, C02F12.8, plk-1, C17F4.7, tag-250, C32D5.8, nspb-12, cpz-1, htp-1, F46B6.5, hrd-1, htp-3, R07B7.2, pqn-59, asp-2, fem-2, W03F11.1, W09D6.5, tag-332, ZK1128.2 | chromosome segregation; mitotic cell cycle; embryonic development |
| 12 | 32 | B0303.7, dyn-1, C17G10.1, C27A12.7, ari-1, C29F7.1, ost-1, C46F11.2, D1037.1, atp-3, F41G4.1, F42H10.5, F53F8.5, F56D2.2, F58F12.1, hid-1, nhr-53, ubc-18, R05G6.10, unc-57, ech-6, T05G5.8, T12E12.1, T14G10.7, Y43B11AR.3, Y54G2A.23, Y55F3AM.10, Y69A2AR.18, tag-349, Y82E9BR.3, sec-24.2, col-19 | ATP synthesis coupled proton transport; zinc ion binding; osmoregulation |
| 13 | 19 | hsp-25, rsd-3, C44B11.1, ras-1, D1081.6, daf-1, aps-1, F32B6.3, F46H5.3, unc-23, M106.4, cyn-11, T20G5.10, ugt-54, W05B10.4, apg-1, Y38C1AA.6, Y39B6A.37, apb-1 | membrane coat; intracellular protein transport; apoptosis |
| 14 | 31 | egl-8, unc-44, dac-1, baf-1, aly-1, C11E4.6, srw-86, spr-2, pdl-1, C37A2.8, arl-3, gon-1, vrk-1, F29G6.1, F35H10.5, bath-41, F40F9.6, unc-119, pqn-53, R07G3.8, act-2, T05E7.1, pqn-70, rde-4, enol-1, twk-40, lem-2, Y43F11A.6, Y48E1C.1, Y48G10A.3, Y59A8B.12 | nuclear membrane organization and biogenesis; regulation of oviposition; reproductive process |
| 15 | 22 | C14H10.3, C56G2.3, rpl-24.1, tag-15, F35A5.4, F46F11.7, nhr-22, M02F4.3, PAR2.1, heh-1, T04D3.1, dpl-1, W08G11.3, efl-1, ttx-1, efl-2, Y54H5A.2, nuo-3, Y65B4A.7, ZC123.4, nck-1, cdc-25.3 | negative regulation of S phase of mitotic cell cycle; cell cycle; gene expresssion |
| 16 | 34 | rbg-3, B0399.2, C01G5.8, rnr-2, rnh-1.3, nasp-1, pkc-2, F07B7.2, pkc-3, tub-1, F10D7.5, F25B3.6, tra-4, F54A3.4, unc-84, bra-1, par-3, F57B10.5, vps-35, lin-53, tag-235, col-77, num-1, vps-26, sptl-3, rnr-1, dnj-23, par-6, egr-1, rpl-7A, clec-76, his-72, gst-39, vps-29 | reproductive process;  ribonucleoside-diphosphate reductase activity; vulval development |
| 17 | 20 | mat-4, rpl-29, C28H8.9, dsh-1, C44C10.4, C44E4.4, hst-1, emb-27, apc-10, kin-14, psr-1, F36G3.1, F42G8.6, rpl-25.1, far-7, T27A3.6, rsp-2, mat-1, Y39B6A.34, Y55B1AL.2 | cell division; ribonucleoprotein complex; embryonic development |
| 18 | 15 | C27H5.4, brc-1, C45G3.5, grd-12, F02E8.4, ubc-9, mei-2, brd-1, K07F5.14, T14G10.4, T19B4.5, tag-237, gei-17, Y57A10A.8, san-1 | ubiquitin ligase complex; condensed chromosome; embryonic development |
| 19 | 16 | C27A12.2, C34C12.2, pqn-20, F07F6.8, hum-2, hmg-1.2, F47G4.4, nmy-1, F59D12.2, M151.7, R119.2, pqn-62, T06D8.3, ima-1, Y37H9A.3, Y66D12A.5 | myosin complex; intracellular organelle part; positive regulation of multicellular organisms growth |
| 20 | 41 | ife-3, C02B8.3, pdi-2, pdi-1, C16C8.4, C31H1.6, C34H4.2, cah-2, srx-137, F11A10.2, F11D5.1, F14E5.2, F21G4.4, F22D6.4, F23C8.5, bch-1, F27D4.1, F47B10.8, F53F8.1, vig-1, pqn-45, inf-1, hus-1, K03D10.3, ceh-26, K12H6.7, ifg-1, hda-3, mom-1, T12D8.6, T14G10.8, egl-20, W09D10.3, Y23H5B.5, Y37E3.11, pqn-85, dpy-18, Y47G6A.14, Y65B4A.1, ZK512.4, ZK899.6 | protein disultide isomerase activity; multicellular organismal development; response to stimulus |
| 21 | 32 | C06H5.2, C08E3.7, C31C9.3, mfb-1, EEED8.3, F02D10.6, fbxa-101, fbxa-88, F25B3.5, F36D1.1, let-92, sdz-18, skr-2, F54D5.9, F59A2.5, cah-3, cogc-6, cogc-8, cku-80, skr-20, T04A8.7, T05H4.2, T10E9.1, W10G11.20, Y105C5B.14, fbxa-116, fog-2, cku-70, Y53F4B.22, cogc-1, Y71H2B.3, pph-4.1 | double-strand break repair; multicellular organismal development; embryonic cleavage |
| 22 | 35 | cyp-34A6, B0507.3, B0511.6, lim-7, mes-6, rfc-3, rfc-1, pmp-3, mec-3, gei-10, F25H8.2, rfc-4, hpr-17, F37D6.2, F42F12.4, F44B9.8, ceh-14, F46H5.7, F53E4.1, mes-3, F54D7.2, rfc-2, ctg-1, K07G5.1, hpl-1, K09A11.1, K12H4.5, dlc-2, mes-2, R07G3.5, lpd-7, T10F2.4, T22H9.1, str-38, csp-1 | DNA replication factor C complex; chromosome; sexual reproduction |
| 23 | 26 | C01C10.2, pqn-11, srx-117, col-125, C34E10.8, C53C7.1, tat-5, F52C6.3, F54D11.2, gfl-1, dpf-7, sma-1, T05F1.4, T06D8.5, zim-3, zim-1, T27F2.2, ekl-4, Y17G7B.2, Y25C1A.6, Y39B6A.11, Y55D9A.2, ZC13.1, ztf-8, npp-10, dpy-30 | embryonic development; body morphogenesis; epigenetic regulation of gene expression |
| 24 | 18 | col-145, kca-1, C34C6.7, arx-6, C56G2.15, hpl-2, arx-2, arx-7, M01E11.2, klc-1, unc-116, T10C6.5, arx-5, Y55F3AM.13, arx-4, arx-1, Y71F9AM.3, arx-3 | actin filament polymerization; cytoskeleton; developmental process |
| 25 | 20 | C06E7.4, C14A4.11, flp-9, rpl-26, F28E10.3, F49C12.14, rpl-25.2, rpl-12, M60.2, gck-1, rpl-11.1, rpl-32, Y37E3.8, Y45F10A.6, teg-1, Y53C12A.4, Y66D12A.9, ZK418.5, rpl-35, ZK792.4 | ribosome; translation; positive regulation of growth |
| 26 | 42 | gst-33, C05C12.4, aos-1, C23H3.3, C25F6.1, tag-251, C52B9.4, acdh-1, mdt-18, unc-15, mig-32, F13D12.3, sad-1, ufd-1, F22H10.3, suf-1, F32D1.9, F37C4.5, npl-4.1, npl-4.2, K08D10.8, K11B4.1, M05D6.2, R06A4.9, msi-1, R144.2, uba-2, mdt-20, bath-40, Y13C8A.1, pap-1, nhr-235, col-71, Y50E8A.9, wee-1.3, tag-276, Y67H2A.1, Y76B12C.7, ndx-8, vrs-2, fzr-1, ZK856.11 | Larval development; response to stimulus; locomotory behavior |
| 27 | 41 | djr-1.1, gei-7, C06A8.6, C13G5.2, C15H11.9, C18A11.1, tag-319, unc-11, C33E10.10, C35E7.1, CC8.2, D2030.6, F10F2.4, bath-30, F18A1.5, tag-93, F25H2.5, gsp-1, cey-1, zyx-1, sex-1, ape-1, dpy-17, gsp-2, F57F4.4, H18N23.2, gfi-2, K05B2.2, M04F3.1, M163.1, R09A1.2, T06C10.3, T09A5.9, chc-1, T23C6.5, T26G10.1, Y113G7B.17, itsn-1, Y32H12A.4, mau-8, wrt-4 | embryonic development; clathrin coat; reproductive process |
| 28 | 18 | C13B9.3, C50F2.3, dbr-1, D2096.2, E01A2.5, pas-5, F37C12.1, hlh-17, F38E11.5, F45G2.4, F53B7.3, F55C5.7, F59E10.3, T14G10.5, cyn-13, Y25C1A.5, Y71F9AL.17, Y92H12A.5 | membrane coat; COPI coated vesicle membrane; protein metabolic process |
| 29 | 24 | ntl-2, unc-75, ntl-4, F21D5.6, F39H2.3, F53B3.3, unc-39, F56D5.5, let-711, K02B9.2, ifc-2, hacd-1, T12B3.4, drr-2, pqn-64, cav-1, bec-1, W03D8.8, ntl-3, ccf-1, gly-8, rps-7, ccr-4, ZK1307.8 | aging; intracellular organelle; cytoplasm |
| 30 | 45 | swp-1, mog-4, mel-11, C13F10.7, taf-13, C24G6.8, trr-1, C54C6.6, rsp-7, tag-184, F23F1.1, gln-5, F26F12.3, rsr-1, taf-5, sig-7, F40A3.6, F40F9.7, F42A9.8, taf-11.1, ctbp-1, dro-1, apm-3, taf-7.1, tre-1, K02D7.2, gln-2, K04H4.5, R02E12.4, R11A8.2, T21C9.12, T26A5.8, mys-1, W06H3.3, taf-6.1, W10D9.4, Y108G3AL.2, Y32H12A.5, taf-2, pcaf-1, Y51H1A.4, Y53F4B.3, Y53H1A.2, Y55B1BR.3, rpa-2 | nucleic acid binding; RNA polymerase II transcription factor activity; glutamine biosynthetic process |
| 31 | 19 | ugt-60, pak-1, C18B2.2, syp-2, C43E11.11, C55A6.9, F18E2.1, F28C10.1, gst-18, F39H12.1, F52E4.7, F57B10.4, F59B1.2, ddl-1, hsb-1, tag-207, bath-43, rga-1, ddl-2 | none |
| 32 | 22 | BE0003N10.3, C14F11.4, hsp-43, C33G3.6, twk-37, col-20, ceh-40, F26F2.3, ceh-20, cpr-4, shc-1, F58G11.6, mek-1, mdt-29, gpd-2, T07C4.3, mdt-27, unc-62, clec-50, ZC581.1, ZK666.8, lec-3 | macromolecule metabolic process; cell migration; cell motility |
| 33 | 26 | C03D6.1, C10C5.4, egl-45, C34E10.10, C37C3.2, D1081.9, D2092.4, F11G11.5, eif-3.G, F25H2.6, F37A4.5, cyn-6, F55A12.10, F57B10.6, ogt-1, R04A9.2, R102.5, R11G1.6, eif-3.C, T27F7.3, W09C5.8, Y54E10A.5, Y71F9AL.14, eif-3.I, ncl-1, ZK370.3 | translational initiation factor activity; embryonic development; larval development |
| 34 | 18 | gna-1, csn-2, B0365.1, C06E2.1, nuc-1, C13G3.3, C40H1.3, C50F4.12, F35D2.4, paa-1, gei-12, F55A12.8, T13H5.4, W03G9.7, csn-3, Y47G6A.22, csn-1, ZK1248.11 | spindle localization; mitotic cell cycle; embryonic development; |
| 35 | 50 | B0272.2, B0361.10, cogc-2, C08B6.8, C15H9.4, C24H12.5, rab-1, cdc-6, C44B7.1, vps-45, bir-2, C50D2.2, ceh-17, snap-1, F08F8.8, rpn-5, F32D8.4, F33H2.6, F36D4.5, F43D9.3, F47G9.1, syn-3, F55A4.1, unc-64, klp-4, rpt-5, F57A8.2, F57F5.1, nsf-1, K02D10.5, cog-1, exo-3, rpn-8, T01G1.3, cdk-1, rpn-9, cyb-3, snb-1, T23G7.3, sel-9, sec-23, cyb-2.1, puf-3, Y54G9A.4, Y57G11C.4, Y60A3A.9, rps-6, syn-16, cyb-1, ZK180.4 | establishment of cellular localization; transport;  oocyte maturation |
| 36 | 30 | B0432.13, C04H5.1, C07A12.7, C18B2.4, C30B5.4, C33C12.9, D2092.1, DH11.2, F20D1.1, syn-13, dpy-11, F52A8.6, swan-1, unc-73, F55F3.3, K01A2.10, K08F11.5, T03G11.6, T20B3.1, W04G5.9, gln-3, sec-8, lys-1, mdt-8, Y67D2.2, Y73B6BL.19, Y73B6BL.30, Y80D3A.9, ugt-3, ZK1127.10 | protein transport; protein localization; small GTPase mediated signal transduction |
| 37 | 50 | B0261.1, icl-1, cel-1, C15H11.8, rpb-2, rgr-1, rpc-1, C48C5.1, D2045.8, psa-4, F09F7.3, F14B4.3, F23B2.13, F25B4.5, F26A3.2, ama-1, F37E3.1, F43E2.2, brf-1, F52C9.7, mnat-1, F59A6.2, H43I07.2, K07C5.6, gst-5, R07E5.3, nex-2, T13H2.5, T13H5.8, snr-3, W01G7.3, W02D9.3, W03A5.4, W09C3.4, Y110A7A.8, snr-1, Y37E3.3, cdk-7, Y46G5A.4, Y50D7A.2, Y54E10BR.6, let-49, mdt-6, Y59A8B.6, snr-7, Y97E10AR.5, ZK1098.1, tag-246, ZK856.10, tag-315 | DNA-directed RNA polymerase activity; embryonic development; positive regulation of growth |
