# Supplementary material for: Information Flow Analysis of Interactome Networks
Source: PLoS Comput Biol. 2009 Apr 10;5(4):e1000350. doi: 10.1371/journal.pcbi.1000350 (PMC2685719; doi:10.1371/journal.pcbi.1000350)
Supplement: Text S1 — In order to better illustrate the properties of information flow which are not exhibited by betweenness, we analyze two toy examples of possible network topologies using either of the two methods. (0.05 MB DOC) [file pcbi.1000350.s013.doc]

**Text S1.** In order to better illustrate the properties of information flow which are not exhibited by betweenness, we analyze two toy examples of possible network topologies using either of the two methods.

Toy Network 1:

In Toy Network 1 all edges (interactions) connecting nodes (proteins) are equally weighted. There are 4 possible pathways between nodes A and B, the shortest one running through node I, the longest through nodes I-F-G-H. Therefore nodes A and B can communicate through multiple pathways. If we use betweenness to find nodes important for A and B to communicate, we can only recover node I, as it is along the shortest path between A and B, A-I-B. All the remaining nodes - C, D, E, F, G, H – score 0 in betweenness.

Unlike betweenness, information flow method considers all possible communication routes between nodes A and B and recovers all participating nodes scoring their importance relative to the length (confidence level) of a given pathway:

C = 0.24

D = 0.16

E = 0.16

F = 0.12

G = 0.12

H = 0.12

I = 1

Note that, since node I participates in all the possible pathways between A and B, it receives a score of 1.

Toy Network 2:

Toy Network 2 consists of two alternative pathways differing by the confidence levels of interactions between the participating nodes. The thicker edges between nodes C, D and D, E indicate higher interaction confidence, *w_1_*, let’s assume it to be 2 times the confidence of the remaining edges, *w_2_*, therefore *w_1_* = 2*w_2_*.

If we use betweenness to find participating nodes in the paths between A and B and weight the edges, we find that we can only recover the shortest path through D (we can assume that the distance measure is inversely proportional to the confidence score *w*). Node F receives a betweenness score of 0. Alternatively, if we decide to weight all the paths equally in order for betweenness to recover the path through F, we are not accounting for the confidence scores and both pathways are treated as equally likely.

If we use information flow, we can recover both pathways between A and B and weight the nodes along them proportionally to the overall path confidence:

C = 1

D = 2/3

E = 1

F = 1/3.

In summary, the above examples illustrate how information flow can find proteins participating in all alternative pathways interconnecting a protein pair. It does so by taking into consideration both the number of proteins along these paths as well as the confidence scores. Such properties are not exhibited by betweenness.
