# Supplementary material for: Information Flow Analysis of Interactome Networks
Source: PLoS Comput Biol. 2009 Apr 10;5(4):e1000350. doi: 10.1371/journal.pcbi.1000350 (PMC2685719; doi:10.1371/journal.pcbi.1000350)
Supplement: Text S2 — We executed the module extraction routines while varying the maximum and the minimum number of proteins allowed in a single subnetwork in order to determine the best size range. (0.06 MB DOC) [file pcbi.1000350.s014.doc]

**Text S2.** We executed the module extraction routines while varying the maximum and the minimum number of proteins allowed in a single subnetwork in order to determine the best size range. We varied the maximum size to be 25, 50, 75, 100 proteins and the minimum size to be 10, 15, 20, 25 proteins. Next, we evaluated GO enrichment among the subnetworks within each size limit combination for a total of 15 combinations (we omitted *<minimum=25, maximum=25>* combination). Below is a plot of the fraction of subnetworks found to be enriched with GO annotations for each minimum and maximum size of subnetworks.

Each line corresponds to a specific maximum subnetwork size (25, 50, 75, 100). The minimum size criteria are satisfied by retaining only the subnetworks whose size is larger or equal to a specific minimum threshold (10, 15, 20, 25).

We can see from the plot that varying the maximum size (corresponding to a single line on the plot) has little effect on the enrichment score. However, as we increase the minimum size requirement, many of the smaller subnetworks are excluded, and the larger remaining subnetworks are more likely to contain groups of proteins sharing functional categories. The majority of the individual subnetworks obtained by varying the upper and lower thresholds are very similar with respect to the genes they contain and therefore GO enrichment.

Each entry in Table* lists the number of subnetworks enriched with GO annotations divided by the total number of subnetworks within each *Min-Max* threshold combination. Each column in the table corresponds to a line in the above plot. For example, the selected threshold combination, 15-50, results in 37 subnetworks of which 35 are enriched in GO categories.

Table *

|  | | **Maximum # proteins**  **in a subnetwork** | | | |
| --- | --- | --- | --- | --- | --- |
|  |  | **25** | **50** | **75** | **100** |
| **Minimum**  **# proteins**  **in a**  **subnetwork** | **10** | 48/63 | 45/56 | 44/54 | 42/52 |
|  | **15** | 34/37 | 35/37 | 34/35 | 32/33 |
|  | **20** | 14/14 | 24/24 | 22/22 | 21/21 |
|  | **25** | N/A | 15/15 | 15/15 | 14/14 |

We selected the 15-50 range for a more detailed analysis as described in the main text because we wanted to keep the overall GO enrichment high while still retaining most of the GO enriched subnetworks. Alternatively, we could have increased the minimum size of the network to be 20 proteins, which would have resulted in all of 24 subnetworks being enriched with GO. However, we would have lost 11 GO enriched modules as compared to 15-50 range.
